# Phytochemical and pharmacological characteristics of phalsa (*Grewia asiatica* L.): A comprehensive review

**DOI:** 10.1016/j.heliyon.2024.e25046

**Published:** 2024-01-19

**Authors:** Simrat Kaur, Rafeeya Shams, Kshirod Kumar Dash, Vinay Kumar Pandey, Ayaz Mukarram Shaikh, Endre Harsányi, Béla Kovács

**Affiliations:** a Department of Food Technology and Nutrition, Lovely Professional University, Phagwara, Punjab, 144411, India; b Department of Food Processing Technology, Ghani Khan Choudhury Institute of Engineering and Technology, Malda, West Bengal, 732141 India; c Department of Bioengineering, Integral University, Lucknow, Uttar Pradesh, 226026, India; d Department of Biotechnology, Axis Institute of Higher Education, Kanpur, Uttar Pradesh, 209402, India; e Faculty of Agriculture, Food Science and Environmental Management Institute of Food Science, University of Debrecen, Debrecen, 4032, Hungary; f Faculty of Agriculture, Food Science and Environmental Management, Institute of Land Utilization, Engineering and Precision Technology, University of Debrecen, Debrecen, 4032, Hungary

**Keywords:** Phalsa, Bioactive properties, Phytochemistry, Health benefits, Antioxidant activity

## Abstract

Phalsa is a tropical and subtropical fruit that is high in nutritional value and is primarily cultivated for its fruit. As, Phalsa fruit contain high number of vitamins (A and C), minerals (calcium, phosphorus, and iron), and fibre while being low in calories and fat. The fruit and seed of Phalsa contain 18 amino acids, the majority of which are aspartic acid, glutamic acid, and leucine. Based on in vivo and in vitro studies phalsa plant possess high antioxidant, anti-inflammatory, anticancer, antimicrobial, antidiabetic properties. However, antioxidant properties are found in the form of vitamin C, total phenolic, anthocyanin, flavonoid, and tannin. The phalsa plant's fruits and leaves have substantial anticancer action against cancer cell lines. Because of the presence of a broad range of physiologically active chemicals, investigations on phalsa plants revealed that some plant parts have radioprotective qualities. The anti-glycosidase and anti-amylase activity of aqueous fresh fruit extract was shown to be substantial. The phalsa plant contains an abundance of biologically active chemicals, allowing it to control microorganisms through a variety of processes. Phalsa methanolic leaf extract was revealed to have antimalarial and antiemetic effects. The hot and cold polysaccharide fractions extracted from the phalsa plant have potent hepatoprotective and therapeutic properties. Therefore, this review is based on the nutritional, bioactive, phytochemicals, and potential pharmacological uses of phalsa. The potential health benefits and economic potential of the phalsa berry's phytochemicals are promising areas for further study.

## 1 Introduction

The fruit berries play a crucial role in routine life and is considered beneficial eatable due to hidden health promoting benefits. Berries contain a number of nutrients that are important for active and healthy lifestyle or entirely serve as an abundant source of energy in the human diet. These berries contain a higher content of bioactive compounds such as anthocyanin, flavonoids, and tannin which are directly linked to various health benefits [1]. Besides, it also encourages health properties through the slowdown in the aging process and minimizes the risk of various diseases including rheumatoid arthritis, cancer, lung disease, cardiovascular disorder. Phalsa *“Grewia asiatica* L.*”* with genus Grewia produces edible fruit [2] which is associated with the family *Tiliacae* and contains about 150 species of short to large shrubs. It is specific to tropical and subtropical areas of the world [3,4]. Phalsa is a flowering plant with small berry fruit conventionally blooms in warm climates and experience leaf failing in wintertime. It is having high nutritional value and is cultivated on a small scale in each state due to the small size of the fruit, repeated harvesting, and prolonged ripening period [5]. Phalsa fruit postharvest performance has received very little research, and suggestions on fruit harvest maturity are required because this factor affects the flavor, customer demand, marketing, and fruit storage life. Second, minimizing post-harvest deterioration is crucial for providing consumers with super-quality products, storage at low temperature is the most effective means of minimizing the metabolic and pathological processes that can cause quality loss [6,7].

Certain acidic fruits are good sources of minerals, vitamins C, and A. It is also rich in phosphorus, and carbohydrate, low in fat, and provide calories [8] and considered to be beneficial in the human diet over the last decades due to the higher content of bioactive compound such as anthocyanin, flavonoids, phenolic compound, tannin [9,10]. When Phalsa fruits are fully mature, they are purple in color and may turn black as they ripen on the bushes. Due to its colored fruit and juice (due to the presence of anthocyanin), phalsa is regarded as an antioxidant in nature. Along with the seeds, the whole fruit is consumed. Fruit with 11–12 % total soluble solids (TSS) and 3 % acidity, makes up 55 % of the fruit juice weight. Fruits that are ripe are used as fresh or in the form of juices. Baskets can be made using the phalsa shoot [11]. Phalsas’ medicinal value is because of the presence of different metabolites including saponins, coumarins, and anthraquinone. The ripe fruit of this plant is used as herbal medicine for stomach upset, diarrhea, intestinal infection, cough, and jaundice treatment [12,13]. Rheumatism is also treated using phalsa roots. Fruits and barks are mainly known for the treatment of household remedies of tissues, wound healing, and osteoporosis and are also regarded as having significant medicinal value [14]. However, phalsa is a perishable fruit with a shorter shelf-life of about 1–2 days after harvest, due to which there is very less time for further processing. So, served as suitable only for local marketing [15,16].

**Fig. 1:**
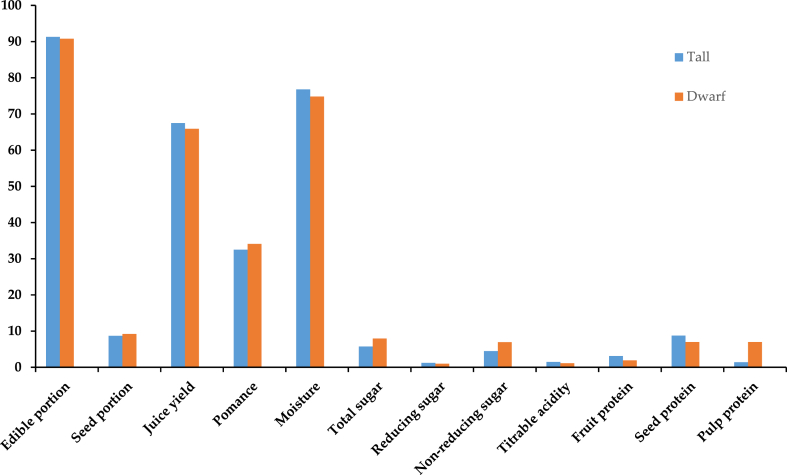
Nutritional proportion of the tall and dwarf type of Grewia asiatic.

In India, two different kinds grown namely tall and short (dwarf) are grown, which differ in terms of various chemical and physical traits viewed in Fig. 1. Juice production is significantly more prominent in the tall variety as it is connected to eatable portions thereby increasing the number of quantities of total sugars and nonreducing sugars found in the dwarf type. When compared to the dwarf type, the tall kind possessed greater seed protein, titrable acidity, and reducing sugars [17]. The delicate nature of fruit berries popularly originates in India and some parts of Southeast Asia including Sri Lanka, Pakistan, and Bangladesh with an inefficient supply chain [12]. The main cultivation areas for phalsa fruit are Gujarat, Bihar, Tamil Nadu, West Bengal, and Maharashtra while this fruit is grown in Rajasthan, Punjab, Uttar Pradesh, and Haryana for commercial purposes [18]. One of the founders of plant physiology Nehemiah Grew gave the name Grewia while Asiatica mirror the Asian origin of this species [19]. In India, each state cultivates phalsa on a very small scale as a minor crop. Only 30 ha of land in Punjab are covered with phalsa, but this yields an annual production of approximately 196 tonnes/hectare [20]. Phalsa is a common plant grown in several regions of South India, including Rajasthan, Uttar Pradesh, Punjab, west Bengal, Haryana, and Madhya Pradesh. Phalsa covers a total area of less than 1000 ha of land. Due to the tiny size of fruit, protracted its period of ripening, repetitive harvesting, and fruits highly perishable nature, the crop's popularity is constrained. Apart from India, it is also grown on an experimental basis in some American provinces, including Pakistan, Vietnam, Bangladesh, Nepal, Sri Lanka, Thailand, and the Philippines [21]. Phalsa has been known by many names such as Phalsa (Hindi, Urdu, Marathi), phulsa (Kannada), Shukri (Gujarati), Unnu (Tamil), Shukri (Bengali) [22].

Phalsa is mostly grown for its fruit, which is eaten raw or added to drinks and foods. The phalsa is a small tree or shaggy shrub that grows up to 12–14 feet high, heart-shaped to the oval shape of leaf with approximately 5–18 cm long, and 9–14 cm broad. Small flowers are arranged in bunches in the leaf forks i.e., in axillary fascicles, each cluster having 2–10 branches i.e., peduncles. It contains edible round shape fruit with a seed inside the flesh. It typically serves the warm climate and leaf loss in winter. The fruit at full ripening possesses dark purple and is covered with very thin whitish layer and becomes tender and soft with depressed spots on the surface with massive and symmetrical housing trichomes [2].

Rich antioxidant content is one of Phalsa's main pharmacological qualities. The fruit is a rich source of flavonoids, polyphenols, and vitamins A, C, and E. Strong antioxidants, these substances counteract damaging free radicals in the body and lessen oxidative stress. This characteristic has a critical role in the prevention of numerous chronic illnesses, such as cancer and heart problems. Phalsa can reduce inflammation [17]. The potential of its extracts to block the body's inflammatory processes has been investigated. This anti-inflammatory action is essential for treating diseases like arthritis and some autoimmune disorders that are brought on by excessive inflammation. Phalsa's antibacterial qualities are also well-known. Research has shown that Phalsa extracts have antifungal and antibacterial properties. This makes it useful for treating illnesses brought on by different microbes in conventional therapy. The possible hypoglycemic (blood sugar-lowering) effects of phalasa have been studied. Phalsa is good for people who have diabetes or are at risk of getting it because it contains compounds that may help control blood glucose levels. The fruit's high dietary fibre content facilitates digestion and supports gut health. It functions as a natural laxative, encouraging frequent bowel motions and avoiding constipation. Because of its cooling properties, phalsa is also used in traditional medicine to treat heat-related illnesses and prevent heatstroke in hotter regions of the world [23]. The objective of this review is to analyze the nutritional profile, bioactive components, phytochemical characteristics, and possible industrial uses of phalsa. This review discusses the phytochemical properties of phalsa, including its pharmacological activities, antioxidant activity, anticancer activity, anti-inflammatory activity, radioprotective activity, anti-diabetes, anti-microbial, anti-malarial, hepatoprotective, *anti*-hyperlipidemic, and analgesic activity.

## 2 Nutritional profile of phalsa

Ripe fruits of phalsa contain a high amount of vitamins A, and C approximately (16.11, and 4.38 mg), minerals (calcium 820.32 mg/100g, phosphorous 814.5 mg/100g, and iron 27.10 mg/100g), and fiber however, low in calories and fat. For a healthy life, each of these elements is necessary [8]. The comprehensive nutrient composition of underutilised fruit, as documented in several studies, is demonstrated in Table 1.

### 2.1 Oil in phalsa

The oil found in phalsa is characterised by its bright-yellow color and contains around 5 % free fatty acid. It has a high concentration of various components, namely oleic acid (13.5 %), stearic acid (11.0 %), linolic acid (64.5 %), unsaponifiable material (3 %), and palmitic acid (8 %), as indicated in Table 2 [24,25]. Aside from it, there are also trace percentages of myristic acid, margaric acid, dihydro malvalic acid, etc. are also present. Phalsa seed is rich/high in triacylglycerol (70.83 %), but low in phospholipids (2.01 %) [25]. Different hydrocarbons and waxes, sterol esters, and triacylglycerols, free fatty acids are all present in phalsa seed oil (1.09 %, 7.91 %, 70.83 %, 2.06 %), different glycolipids (2.50 %), and phospholipids (2.01 %) are also present. Along with tocopherol, phalsa seed oil also includes α-tocopherol (651.35 mg/100 g), β-tocopherols (5.01 mg/100 g), and γ-tocopherols (1.08mg/100 g). Contrasting tocopherols present suggest that phalsa has potent anti-inflammatory capabilities whereas, the sterol profile shows the presence of β-Sitosterol, fucosterol, and stigmasterol (18.30 mg/100 g, 2.91/100 g, 4.04 mg/100g). Fucosterol. Campesterol, fucosterol, and cycloartenol are all found in the oil at a trace level [3].

**Table 1:** Nutritional constituents of phalsa fruit.

Nutrients	[26] (Georgia, port valley)	[8]	[27]
Calories (Kcal)	90.50	–	72.00
Moisture (%)	76.30	–	76.30
Fat (g)	<0.10	0.90	0.10
Protein (g)	1.57	1.30	1.60
Carbohydrates (g)	21.10	14.70	21.10
Dietary fiber (g)	5.53	1.20	5.53
Ash (g)	1.10	1.10	–
calcium (mg)	136.00	129.00	136.00
Phosphorus (mg)	24.20	39.00	–
Iron (mg)	1.08	3.10	1.08
Potassium (mg)	372.00	–	372.00
Sodium (mg)	17.30	–	4.40
**Vitamins (mg)**			
Vitamin C, Ascorbic acid	4.385	22.00	4.38
Vitamin A	16.11	419.00	–
Vitamin B1, Thiamine	0.02	–	0.03
Vitamin B2, Riboflavin	0.264	–	0.01
Vitamin B3, Niacin	0.825	0.30	–
**Minerals (mg)**			
Iron (Fe)	–	–	140.80
Copper (Cu)	–	–	0.48
Zinc (Zn)	–	–	1.44
Nickel (Ni)	–	–	140.80
Cobalt (Co)	–	–	0.99
Chromium (Cr)	–	–	1.08

**Table 2:** Oil composition in phalsa.

Oil	Composition (%)
Oleic acid	13.5 %
Stearic acid	11 %
Linolic acid	64.5 %
Unsaponifiable material	3 %
Palmitic acid	8 %

### 2.2 Proteins and amino acids

Phalsa fruit and seed indicate the symptoms of both non-essential amino acids and essential amino acids [28]. Phalsa fruit and seed have 18 different amino acids with the maximum concentration of aspartic acid (19.06 %), glutamic acid (11 %), and leucine (11.02 %) [29]. Amino acid is the main indicator for accessing the quality of protein. Whereas taurine, serine, and phosphoserine is the dormant amino acid present in phalsa juice and a large amount of aspartic acid, threonine, tyrosine, and glycine [8]. The fruit pulp contains more concentration of phosphoserine in contrast to all other free amino acids. Essential amino acids are found to be in an excellent concentration in the peel and pulp. Like: isoleucine (4.4 %), threonine (3.3 %), arginine (9.5 %), and phenylalanine (3.8 %). Moreover, the fruit of *G. Asiatica* has a high content of histidine, threonine, and lysine [30].

### 2.3 Vitamins

Phalsa fruit contains numerous amounts of vitamins. Vitamin C content present in phalsa is around (4.38 mg/100g) which promotes the synthesis of collagen, aids in wound healing, and enhances the absorption of iron from food. Vitamin A (16.11 mg/100g) is a natural nutrient present in fruit that helps to promote good vision and prevent macular degeneration. Vitamin B1 with (0.02 mg/100g) helps to maintain heart and nerve function. While, vitamin B2 (0.26 mg/100g), and vitamin B3 (0.82 mg/100g) are also present to some extent [8].

### 2.4 Minerals

Phalsa fruit contains a sufficient amount of minerals it helps in monitoring synthesis along with the smooth flow of blood such as sodium (17.30 mg/100g), potassium (372.00 mg/100g), calcium (136 mg/100g), chromium (0.48 mg/100g), zinc (1.44 mg/100g), iron (140.80 mg/100g), nickel (2.61 mg/100g), phosphorus (24.20 mg/100g), cobalt (0.99 mg/100g). A great source of sodium, well it acts as an electrolyte and essential ion to promote enzyme operation and muscle as well and is necessary for blood regulation. The presence of potassium and protein builds good muscle and strengthens the muscle [8,27]. Total ash, ash soluble in acid, and ash soluble in water were 3.0, 1.4, and 1.1 %, respectively. A sufficient intake of fiber reduces the risk of obesity, cardiovascular disease, diabetes, and several types of cancer. Phalsa was analyzed for six micronutrients: Iron, copper, zinc, nickel, cobalt, and chromium on actual fresh weight and dry weight [27]. The pH value of phalsa fruit that is acidic in nature ranges from 2.7 to 3.3.

### 2.5 Carbohydrates

The carbohydrate level in phalsa fruit is around 21.10g which constitutes starches, and sugars. It is the primary source to provide energy for proper functioning and physical activity. The fruit has a sugar concentration of 7.95 % total sugar, 0.99 % reducing sugar, and 6.96 % non-reducing sugar [2]. Due to its rich source of reducing sugar as it imparts a sweetening effect of about 10–14 % and specifically lowers the risk of obesity and overweight. A sufficient amount of dietary fiber with 5.53 g/100g contains soluble and insoluble fiber that reduces the risk of obesity, cardiovascular disease, diabetes, and several malignancies.

## 3 Bioactive components of phalsa

Phalsa is a rich source of bioactive compounds namely anthocyanin, flavonoid, and tannin content. However, the concentration of these compounds depends upon different factors such as growing conditions, time of harvest, post-harvest storage, fertilizers used during extraction, the season of growing, and agronomic practices employed. The phytochemical study focused on screening fruit for anthocyanin, flavonoids, phenolic acid, vitamin C, and carbohydrate while the seed contains tannin, starch, minerals, and oil [13]. These flavonoids, phenolic content ((4.608 QE mg/g), (144.11 mg GAE/g)), and anthocyanin content (4.882 mg/kg) were observed to have a considerable amount of antioxidant activity [31].

### 3.1 Anthocyanin

**Fig. 2:**
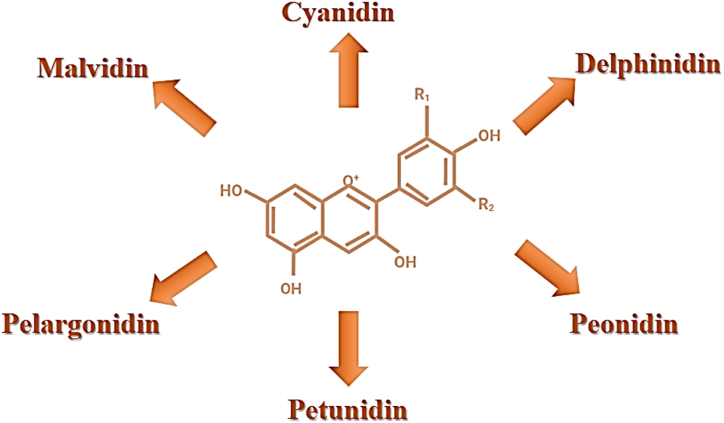
Categories of anthocyanin (i) Cyanidin (R_1_: OH, R_2_: H), (ii) Delphindin (R_1_: OH, R_2_: OH), (iii) Pelargonidin (R_1_: H, R_2_: H), (iv) Peonidin (R_1_: OCH_3_, R_2_: H), (v) Petunidin (R_1_: OCH_3_, R_2_: OH), (vi) Malvidin (R_1_: OCH_3_, R_2_: OCH_3_).

Anthocyanin is a member of the flavonoid group with water-soluble vacuole pigments present in plants i. e, fruits and vegetables. Depending on pH they give unique colors to plant and plant parts like red, purple, and blue color of various botanic origin [32]. Anthocyanin is available as a glycoside with respective gylcone and derivative glycosylated [33]. There are several types of anthocyanin, however these may be generically classified into six categories: pelargonidin, delphinidin, petunidin, peonidin, malvidin, and cyanidin [34]. The six categories of anthocyanin are illustrated in Figure (2). The present study of phalsa revealed that the phalsa is a promising indigenous fruit rich in a total of seven anthocyanins with cyanidin-3-O (6″-acetylglucoside) as an abundant amount of 44–63 %, pelarigonidin-3-*O*-(6″-acetylglucoside), peonidin-3-*O*-glucoside with (8–14 %) and (3–30 %), malvidin-3-*O*-glucoside pyruvic acid, delphinidin-3-*O*-glucoside, pelarigonidin-3-*O*-malonyl glucoside, peonidin-3-*O*-(6″-acetyl glucoside) [10]. Additionally, by assessing total anthocyanin, it has been determined that extract in phalsa fruit contains a high amount of anthocyanin and that it has potent antibacterial properties as opposed to four separate gram-negative and gram-positive species. The dearth of literature on the plant mentioned shows that, despite its wide range of applications, it has been treated with astonishing indifference. It was important to further the research into the fruit pigment in order to enhance the commercialization of phalsa fruit goods. The focus of the current work is on the identification and quantification of unique anthocyanins from phalsa fruit, taking into consideration their therapeutic potential relevance and potential industrial application as a natural colorant [34]. Also noted is its solubility in various solvents and stability at various temperatures when exposed to light and darkness.

### 3.2 Flavonoid

Flavonoid belongs to the class of polyphenolic (secondary (2⁰) metabolites) found in relatively all vegetables and fruits. Flavonoids are typified by 15-carbon atoms consisting of a one-heterocyclic ring and two-phenyl rings [35]. Flavonoids are almost available in plants in free aglycone and glycoside bonds. The glycoside bond found is the most common flavonol and flavone consumed in the human diet [36]. Total flavonoid content present in phalsa callus, stem, barks, leaf, etc. Generally; flavonoids are divided into a number of different groups, six of which are anthocyanin, flavones, isoflavones, flavanones, flavan-3-ols, flavonols [37]. Executed research on flavonoid content by Ref. [14] in different parts of phalsa extract obtained from stem, and leaf according to vivo and vitro studies. Flavonoid content in phalsa pomace was assessed to be 12.42 ± 0.56 (CE mg/g) [38,39] determined in rind/bark 39.11 ± 4.65 mg of flavonoid content leaf extract have highest amount with quercetin in the quantity of 4.28 ng/ul is observed over dry matter. While the other study revealed that the solvent extract of fruit shows total flavonoid content (GAE) and phenolic content to be 0.13 and 5.25 respectively [40].

### 3.3 Tannin

Tannin is a soluble astringent complex of polyphenolic biomolecules (secondary metabolites) containing sufficient amount of hydroxyl and carboxyl to form strong complex with other macromolecules. These are widely distributed in plant origin whereas, phalsa contains tannin in countless amounts in leaves, stems, fruit, root, bark, and seed [[41], [42], [43]]. The tannin content in phalsa pomace is recorded as 0.5 ± 1.25 (g/100g) by Gupta et al. [38] while Elhassan & Yagi [28] reported that the total tannin in phalsa fruit is 1.13–2.46 %. In phalsa fruit, it seems that methanol and acetone extract in tannin inhibit the growth of micro-organisms. Other studies assume that tannin amount in the leaves, stem, and bark inhibit various micro-organism and therefore, helps in various disease prevention cancer, cardiovascular, kidney diseases, and diabetes [9,44,45]. Table 3 provides comprehensive information regarding the potential impact of bioactive substances on human nutrition.

**Table 3:** Role of bioactive compounds in human nutrition.

Bioactive Component	Role in Human Nutrition	Functional Properties	Applications	References
Antioxidants (Vitamin C, Polyphenols)	Protects cells from oxidative damage, supports immune system, may reduce chronic disease risk	Antioxidant, anti-inflammatory, anti-cancer	Dietary supplements, functional foods	[46]
Dietary Fiber	Aids digestion, promotes bowel regularity, supports weight management	Digestive health, satiety	Fiber supplements, bakery products	[47]
Phenolic Compounds	Antioxidant, anti-inflammatory, potential anti-cancer effects	Antimicrobial, anti-inflammatory	Nutraceuticals, herbal medicines	[48]
Minerals (Calcium, Iron, Potassium)	Essential for bone health, blood circulation, nerve function	Electrolyte balance, enzyme cofactors	Fortified foods, mineral supplements	[49]
Carotenoids (Beta-Carotene)	Precursors to vitamin A, important for eye health	Antioxidant, vision support	Nutritional supplements, baby foods	[50]
Tannins	Astringent taste, potential antimicrobial effects	Antioxidant, anti-inflammatory	Traditional medicines, food preservation	[51]
Phytochemicals (Quercetin, Kaempferol)	Antioxidant, anti-inflammatory, potential anti-cancer effects	Antimicrobial, immune support	Herbal remedies, dietary supplements	[52]
Vitamins (B-Complex, Vitamin K)	Essential for energy metabolism, blood clotting, and overall health	Coenzymes, bone health	Nutritional supplements, fortified foods	[53]
Omega-3 Fatty Acids	Heart health, cognitive function	Brain development, anti-inflammatory	Fish oil supplements, functional foods	[54]
Protein	Building blocks for tissues, enzymes, and hormones	Muscle repair, enzyme function	Protein supplements, sports nutrition	[55]

## 4 Phytochemical properties

Phytochemicals expose the presence of primary metabolites namely essential amino acids, mucilage, alkaloids, saponins, glycosides, steroids, fixed seed oil, and alkaloids [56], while this fruit has been found to contain a variety of secondary metabolites, including myricetin, naringenin, cyanidin, pelargonidin, and hydroxymethylfurfural, hydroxybenzoic acid [57]. Aqueous extract or other solvents such as petroleum ether, methanol, distilled water, ethyl acetate, and benzene contain (1.2 %, 13.6 %, 12.5 %, 1.5 %, 1.3 %) are used to extract these phytochemicals. Pharmacognostic estimates 5 % ash along with acid-insoluble ash 2.1 % and water-soluble ash 2.5 % in leaves. Moreover, phytochemical screening of fruit in methanolic extract shows the presence of carbohydrates, tannins, phenolic compounds, vitamin C, and flavonoids. While fixed oil and flavonoid in ethyl acetate, petroleum ether extract, and aqueous extract contain tannin, carbohydrate, and phenolic [19]. In addition; ethanol extract from fruit contains different amino acids namely proline, glutaric acid, lysine, phenylalanine, and carbohydrate such as xylose, and arabinose. Under UV (254 and 366 nm) and visible light, fluorescence properties of the powdered leaves and extracts were seen [56,58].

**Fig. 3:**
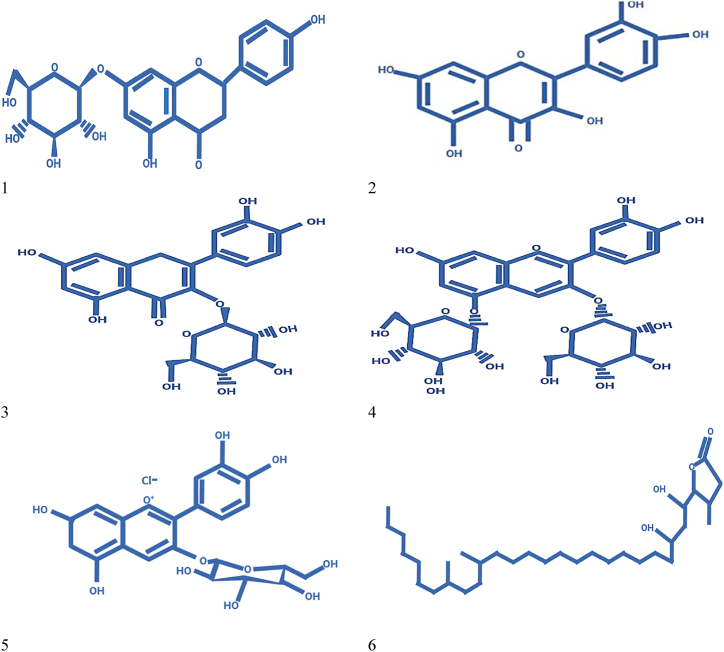

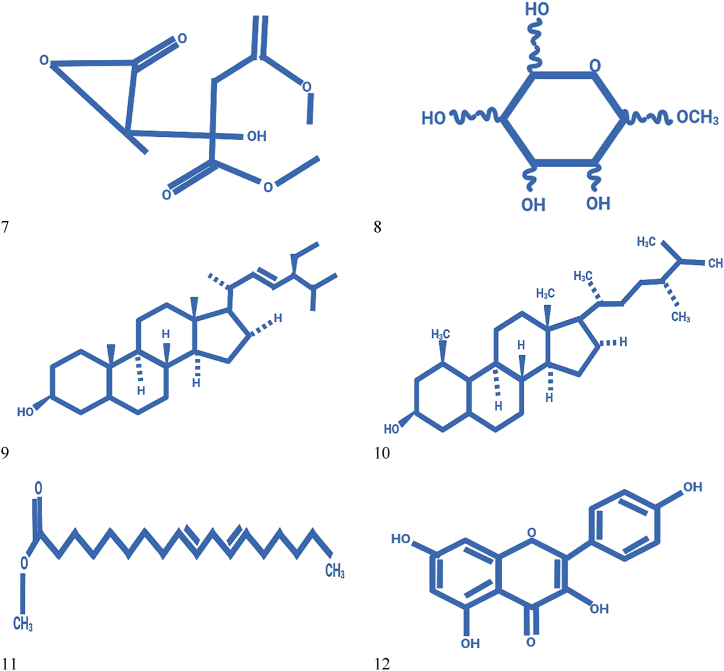

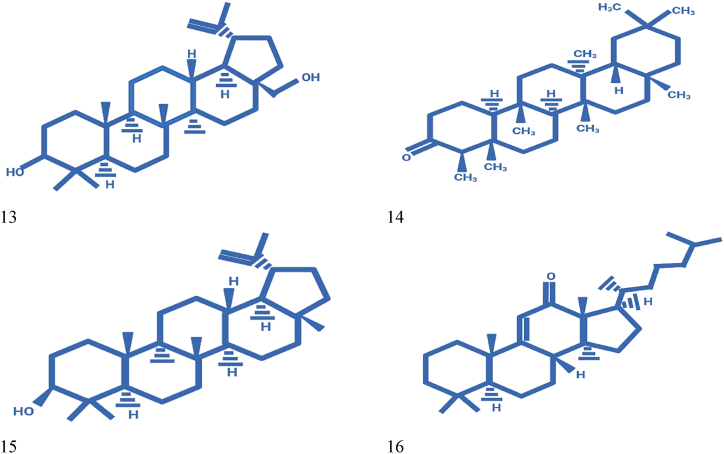
Secondary metabolites isolated from G. asiatica: (1) Naringenin -7-*O*-β-d-glucoside, (2) Quercetin, (3) Quercetin 3-*O*-β-d-glucoside, (4) Pelargonidin-3,5-diglucoside, (5) Cyanidin-3-glucoside, (6) δ-lactone 3,21,24-trimethyl-5,7-dihydroxy-hentriacontanoic, (7) Citric acid trimethyl ester, (8) α-methyl-*l*-sorboside, (9) Stigmasterol, (10) Campesterol (11) 9,12-octadecadienoic acid methyl ester, (12) kaempferol, (13) Betulin, (14) Friedelin, (15) Lupeol, (16) Lanost-9 (11)-en-12-one.

While a range of secondary metabolites such as cyanidin-3-glucoside, naringenin -7-*O*-β-d-glucoside, pelargonidin-3,5-diglucoside, quercetin, quercetin 3-*O*-β-d-glucoside are the blend that are confined from fruit. Similarly, quercetin 3-*O*-β-d-glucoside, pelargonidin-3,5-diglucoside, quercetin, δ-lactone 3,21,24-trimethyl-5,7-dihydroxy-hentriacontanoic acid and naringenin -7-*O*-β-d-glucoside present in flower [59]. Phalsa pomace contains main compounds namely campesterol, α-methyl-*l*-sorboside, stigmasterol, citric acid trimethyl ester, and 9,12-octadecadienoic acid methyl ester [60]. Leafy compounds high in kaempferol, quercetin, and a combination of their glycosides. Betulin, lupeol, lupenone, and friedelin are additionally present in stems and bark [61,62]. Structures of secondary metabolites and isolated chemicals from phalsa fruit are displayed in Fig. 3.

## 5 Pharmacological activities of phalsa

Phalsa is a plant that has been shown to have a variety of beneficial impacts on human health and is regarded as being both nutritionally and therapeutically significant [13]. Various studies carried out over the last years to investigate the pharmacological and dietary benefits of the phalsa fruit plant [2,62]. Phalsa fruit has also been used as a folk treatment for a variety of ailments. Inflammatory conditions as well as respiratory, cardiac, and blood diseases. Different polyphenolic components of the phalsa plant were found to have significant antioxidant activity (flavonols, phenolic acids, and anthocyanins). The pharmaceutical business is observing a change in the effectiveness of drug consumption, with synthetic pharmaceuticals being substituted with natural plant-based solutions more frequently due to consumers' growing opposition to medical aliments to drug-based treatment [63]. Due to their high phytochemical content, plant-based changes to synthetic medications are thought to have improved disease-preventing abilities. Phalsa fruit has traditionally been utilized for its radioprotective, anti-inflammatory, anti-microbial, and anticancer properties, including the treatment of heart disease as shown in Table 4.

**Table 4:** Pharmacological effect of Phalsa.

Pharmacological activities	Mode of action	Reference
Antioxidant	The fruit exhibits free radical scavenger's activity with different fractions, inhibit DPPH, FRAP, ABTS and isolation of phenolic content.	[43,58,64,65]
Anticancer	The fruit and leaves inhibit five different cancer cell lines NCI–H522, MCF-7, HEK-293, HELA, HEP-2. Methanolic extract of phalsa increases the life expectancy of (EAC) Ehrlich's ascites carcinoma tumor bearing mice and inhibit chronic myelogenic leukemia (K-562), breast adenocarcinoma (MCF-7), severe myeloblastic leukemia (HL-60), cervical epithetlial carcinoma (HELA) with IC₅₀.	[66] [38] [66] [67]
Anti-inflammatory	Orally administration of methanolic extract of phalsa constitutes reduce swelling (p < 0.01) carrageenan-induced edema rat paw. The hydrolyzed polysaccharides from phalsa leaves have an anti-inflammatory impact on adult albino rats with paw edema. Methanol and water used as solvent for extraction from dried roots.	[68] [69]
Radioprotective	The amount of spermatogonia “A,” spermatogonia “B", spermatid, and spermatocytes results were remarkably higher in mice that are treated with phalsa extract over a 30-day period in contrast to the standard group of Phalsa. Mice were slaughtered at various time interval (1, 3, 7, 15, 30 days) and the cerebrum were examined to determine levels of lipid peroxidation (LPO), proteins, and glutathione (GSH). Based on various groups: group 1, Group 2, group 3, group 4. Mice was treated with different doses of supplement for 15 consecutive days under 5 Gy of gamma radiation.	[70] Ahaskar & Sharma (2006)
Anti-diabetes	Extraction of phalsa plant show effect on STZ-induced hypergylcemic rats and liver glycogen, serum glucose, superoxide dismutase, reduced GSH were administrated orally to mice. Methanolic extract of leaves decreased the blood glucose level in treated alloxan induced diabetic wister rats. And inhibit α-amylase and α-glucosidase Stems, leaves, and fruit of the phalsa plant stimulate insulin, antioxidant activity, and radical scavenging.	[71] [72] [40] [73] [59]
Antimicrobial	Methanol extract of phalsa pulp and peel used to isolate polyphenolics content which was furthermore divided into groups: neutral fraction A, neutral fraction B to analyzed antimicrobial effect. Gram-positive bacteria are more susceptible to crude extract and bioactive constituents when exposed to phalsa pomace extract than gram-negative bacteria.	[64] [60]
Anti-malarial	Phalsa leaves exhibits antimalarial activity and emetic action of about 69 % and 59.69 %. Crude alcoholic extract in phalsa fruit was found non-toxic in mice and cause antiemetic effect in dogs	[61] [74]
Anti-hyperlipidemic	Phalsa leaves consist of strong antihyperlipidemic action and low in cholesterol level. Methanolic extract of phalsa fruit lowers the level of triglyceride, cholesterol, blood coagulation, low density lipoprotein. Methanolic extract in bark of phalsa plant reduces triglyceride, total cholesterol, increase in high density lipoprotein and very low-density lipoprotein in rats	[75] Debajyoti et al. (2012) Khatune et al. (2006)
Analgesic activity	Phalsa root bark was tested using an Eddy hot plate and an acetic acid-induced writhing test with aqueous extract showed stronger analgesic effect as compared to methanolic extract.	[68]

### 5.1 Antioxidant activity

Phalsa contains high content of antioxidants including Vitamin C, total phenolic, anthocyanin, flavonoid, and tannin [58]. Radical scavengers of different fractions are anthocyanin, flavanols phenolic acid, and flavonols. The role of polyphenols in fresh phalsa samples have potent in vitro antioxidant activity which is involved in heart diseases (hepatitis and Mellitus). More recent research [43] from earlier studies observed that the antioxidant content in freeze dried fruit is higher than the fresh form and also these freeze-dried fruits have high levels of total phenolic and flavonoid content (294.453 ± 4.696 mg GAE/g, and 116.96 ± 10.71 mg GAE/g) respectively. Polyphenolic content was separated from crude methanol extract of phalsa peel and pulp. The extract is further divided into two different proportions anthocyanin, and flavanols proportion. While the study by Ref. [31] explained that the antioxidant properties of a methanol extract with a higher amount of total phenolic acid, flavonoid content, total anthocyanin content (144.11 mg GAE/g, 4.608 QE mg/g, 4.892 mg/kg) as compared to another solvent. Other parts of the phalsa plant also vary in antioxidant properties as pomace of phalsa fruit evaluate tannin, saponins, flavonoids, and alkaloids content compared to dry matter were 0.52 ± 1.25 g/100g, 1.05 ± 0.96 g/100g, 12.42 ± 0.56 mg/g, and 1.56 ± 1.2 g/100g respectively. These results indicates that even the waste pomace contain significant amount of antioxidant properties. Significantly the antioxidant activity of storage fruit decreases, seed size reduces from 49.0 to 19.4 TEAC mol/g, pulp from 56.1 to 25.8 TEAC mol/g, and peel size 87.8 to 33.13 TEAC mol/g. The aqueous extract of phalsa fruit comprises flavonoid content (5.25 GAE/g) and phenolic content (0.13 GAE/g) respectively. So, researchers suggest the factors that the antioxidant activity in phalsa (anthocyanin, total tannin, total phenolic, total flavonoid content) depends on its growing period/conditions (climate and soil), fertilizer used, its maturity, post-harvest condition, storage conditions, season and solvent used for extraction [45,69]. Other components of the phalsa plant such as flower and bark have similar high antioxidant content [39,45]. Whereas, roots have high antioxidant content of about ABTS (96.42 ± 2.17 %), DPPH (82.6 ± 5.66 %), FRAP (82.53 ± 3.16 %) [65].

### 5.2 Anticancer activity

Fruits and leaves of the phalsa plant inhibit significant anticancer activity against cancer cell lines. Methylthiazolyl tetrazolium (MTT) determined vitro cytotoxin/anticancer activity makes use of Breast cell line MCF-7, epidermal Kidney cell line HEK-293, Cervical cell line HELA, Laryngeal cell line HEP-2, Lung cell line NCI–H522. Fruit extract can be found to be effective on the breast (MCF-7) (IC₅₀ = 58.65 μg/ml) and lung (NCL-H522) (IC₅₀ = 59.03 μg/ml) cancer cell line, moreover, the extract of the leaf will be significantly active at Laryngeal (HEP-2) (IC₅₀ = 61.23 μg/ml and breast (MCF-7) (IC₅₀ = 50.37 μg/ml) [66]. All this revealed that leaves and fruit extract are considered important against the balancing of human cancer. The vitro cytotoxic and vitro antitumoral activities of methanol extract of phalsa leaf against Ehrlich's ascites carcinoma (EAC) cells were assessed by methylthiazolyl tetrazolium assay against four human cell lines; HELA, MCF-7, HL-60, K-562 with 50 % (IC₅₀) values of 177.8, 199.5, 53.70, 54.90 μg/ml respectively [76,77]. Phalsa with a concentration of 250–500 mg/kg methanolic extract to male swiss albino mice maximize the life expectancy of (EAC) Ehrlich's ascites carcinoma tumor bearing mice is 41.22 %–61.06 % respectively. It was known to be that fruit abolished the breast cancer cell line except for cervical (HELA), and laryngeal (HEP-2) cancer cell lines [66].

### 5.3 Anti-inflammatory activity

Inflammation is caused when physical factors trigger an immune system [78]. Phalsa fruit contains a strong anti-inflammatory effect, and screened with methanolic extract in fruit (250 mg/kg and 500 mg/kg) orally and show significantly (p > 0.1) which reduces lump in carrageenan-induced edema in the rat paw. Phalsa plant roots that is dried and then extracted subsequently using methanol and water as a solvent (200 mg/kg and 400 mg/kg) orally and shows significant (0.1 ml 1%wt/vol) to carrageenan-induced edema in rat paw and under positive control (indomethacin 19 mg/kg) [68]. All this reported that changes after 3 h the methanolic extract (59.14 %) of roots of phalsas’ shows higher anti-inflammatory content in comparison to aqueous extract (53.04 %). So, it is examined that methanolic extract shows stronger anti-inflammatory activity than aqueous extract. When given orally to adult albino rats with Paw edoema, hot hydrolyzed polysaccharides and cold hydrolyzed polysaccharides (82 %, (69 %) had an anti-inflammatory effect [69].

### 5.4 Radioprotective activity

Despite its favorable advantages, nuclear radiation is being used more frequently for human welfare, so it is necessary to look into its side effects. Radiation biology's main concern is the search for chemical compounds that can shield people from ionizing radiation [79]. The mice were separated into groups: group 1 get no treatment, group 2 received a daily supplement dose of 700 mg/kg for 15 days, and group 3 (control) accept distilled water administered orally for 15 days similar to the extract before being exposed to 5 Gy of gamma radiation and group 4, to which the extract was supplied orally for 15 consecutive days, and exposed to a single dosage of 5Gy of gamma radiation, once every day [80] Mice sacrifices were made at various elevation times (1, 3, 7, 15 and 30 days). Brains were taken out to estimate glutathione (GSH) and Lipid peroxidation (LPO). Previously in vitro and in vivo studies of phalsa plants showed certain plant sections had radioprotective properties because of the presence of a wide variety of physiologically active substances. When tested on mice, phalsa fruit pulp powder showed the radioprotective effect. The dose administered to the mice was 700 mg/kg bw for fifteen days. In addition to lowering LPO and raising GSH (glutathione) levels in the brain of mice, the fruit pulp powder also shielded the test mice's brains against oxidative damage [80]. Phalsa extract has been shown to have potential as a radiation exposure prophylactic in other in vitro and in vivo experiments. In mice brains, extract of phalsa fruit (700 mg/kg bw) dramatically decreased GSH and LPO levels as compared to controls, according to a study by Sharma & Sisodia [81]. In addition, the extract demonstrated potent radical scavenging and radioprotective properties in protein carbonyl tests. The number of spermatocytes, spermatogonia “A,” spermatogonia “B,” and spermatid count were basically higher in mice that are treated with extract over a 30-day period as compared to the command group in a subsequent by Sharma & Sisodia [82]. Its conclusion is that phalsa fruit extract is also likely to protect mice testes from radiation. This was again supported by Singh et al. [70]; that demonstrated the phalsa fruit shield tested mice's blood against radiation-induced changes.

### 5.5 Anti-diabetes

A hereditary condition known as diabetes mellitus causes blood sugar levels to consistently rise below 7.0 mmol/L (126 mg/dl) (WHO, 2006). Polydipsia, polyphagia and Polyuria, polyphagia, polydipsia, and weight reduction are all signs of the illness. By 2030, diabetes mellitus is expected to overtake smoking as the seventh largest cause of death globally, according to WHO (Bennett et al., 2018). Amazingly, according to the International Diabetes Federation, there were 382 million documented cases of diabetes worldwide, and that number will rise to 592 million by the year 2035. Among them, 65.1 million persons with diabetes in the 20- 79-year age range live in India alone. India is sitting on a diabetes volcano as it approaches numbers that are expected to be reached by 2030 [83] and research indicates that diabetes and hygiene are linked to 2.6 % of all health problems. However, multiple studies have found that some plants, including the phalsa plant, contain antioxidant and phytonutrient qualities that may be useful in the treatment of the disease [84].

Additionally, antihyperglycemic action was observed in the fruit, stems, and leaves of phalsa plant in a study [71]. Most solvent extracts (aqueous, chloroform, methanolic, butanol, ethyl solvent) were orally administered of about (100 g/kg) alloxan-induced hyperglycemic rabbits (up to 200 mg/kg bw) for a span of 24 h. It can be depicted from the study that every extract decreased the triglycerides, blood glucose, and blood cholesterol levels over ground herbal drugs of phalsa bark in alloxan and normal induced diabetic rabbits, the result was greatest for the water-based extract. It's interesting to note that the phalsa fruit has greater blood glucose lowering effects than the bark and leaves of the plant. According to Khattab et al. [72]; this antihyperglycemic was brought on by a number of causes, including insulin, stimulation, antioxidant activity, and radical scavenging. Streptozotocin (STZ)-induced hyperglycemic rats, the phalsa fruit was used for the screening of antihyperglycemic properties. Rats received oral administration of a phalsa fruit methanolic extract (100–200 mg kg–1 day–1 bw) for 28 days, during which time decreased GSH, liver glycogen, serum glucose, superoxide dismutase, and malondialdehyde activity assessed by Khattab et al. [72]. In a different study, Patil et al. [59] described the antihyperglycemic efficacy of phalsa leaf extract in alloxan-induced diabetic Wister rats. When compared to the standard control treatment (glibenclamide 10 mg/kg bw), the study showed that the methanolic parts of leaves (200 mg/kg bw) decrease the glucose level in blood-treated alloxan-induced diabetic wister rats. Moreover, fresh phalsa fruit was shown to have an inhibitory effect against α-amylase and α-glycosidase by Ref. [5]. Aqueous fresh fruit extract was found to have strong anti-glycosidase (IC50 0.41 mg/ml) and weak anti-amylase (IC50 8.93 mg/ml) activity. Hyperglycemia in pancreatic β-cells causes metabolic disturbances that lead to diabetes mellitus, a chronic illness. Type 1 diabetes mellitus, or insufficient insulin synthesis by the pancreas, or Type 2 diabetes mellitus, or insufficient insulin production in the face of insulin resistance, can both result in hyperglycemia [23]. The antidiabetic properties of phalsa fruits were further proven in a vitro work [38] which revealed that the pomace of the fruit had a powerful antidiabetic impact and suggested possible uses for the fruit in the treatment of diabetes. The study's findings demonstrated that aqueous acetone extracts (80:20 vol/vol) had superior anti-diabetic effects than aqueous methanol extracts (80:20 vol/vol) and extracts from mixed solvents, such as water, ethanol, and hexane (10:80:10 vol/vol) extracts. Mixed solvents (85.20 mg/ml), methanol (45.70 mg/ml), and acetone (138.10 mg/ml) were the three extracts with the highest antidiabetic effects (measured by alpha-amylase inhibition). Another investigation into the phalsa plant's leaf extract discovered that phalsa leaf extract (200 mg/kg and 500 mg/kg), when administered orally for 21 days to streptozotocin-induced and control rats (50 mg/kg bw), decreased blood glucose levels in a dose-dependent manner and also increased the animals' glucose tolerance [85].

### 5.6 Anti-microbial

Anti-microbial used for the treatment of pustular eruptions and skin rashes, phalsa leaves are employed because they have antimicrobial activity. The phalsa plant has an abundant amount of biologically active substances that enables it to suppress of bacteria from a number of different mechanisms. The phalsa plant leaves’ ethanolic extract can inhibit 9 different fungal strains, including *Macrophomina phaseolina, Aspergillus effuses, Aspergillus parasiticus, Trichophyton rubrum, Aspergillus niger, Candida albicans, Yersinia aldovae, Saccharomyces cerevisiae*, and *Fusarium solani* according to research the use of agar-disc diffusion method. Eight harmful bacterial species, including *Salmonella typhi, Micrococcus luteus, Escherichia coli, Bacillus subtilis, Pseudomonas aeruginosa, Proteus mirabilis, Staphylococcus aureus,* and *Citrobacter species*. appear to be inhibited by the extract as well [86]. Similar findings were found in another study by Ref. [87]; in which the writers showed that the leaves of the phalsa plant had potent non-bacterial activity against antifungal activity against yeast (*Saccharomyces cerevisiae*), gram-positive bacteria (*Bacillus subtilis*), gram-negative bacteria (*Pseudomonas aeruginosa*). According to other studies testing the bark and fruit of phalsa plants against a variety of bacterial strains (*Salmonella typhi, Bacillus subtilis, Proteus vulgaris, Streptococcus pneumoniae, Staphylococcus aureus, Shigella dysenteries, Staphylococcus epidermidis, Escherichia coli, and Proteus mirabilis*), only three of the bacterial strains tested exhibited strong inhibitor *Escherichia coli, Proteus vulgaris, and Staphylococcus epidermidis* all recorded inhibitory zones of 6.51 ± 0.40 mm, 6.33 ± 0.48 mm, and 7.33 ± 0.85 mm, respectively [88].

Phalsa pulp and peel crude methanol extracts were used to isolate polyphenolics into a portion of ethyl acetate. Additionally, three groups are formed: neutral fraction A, which contains additional flavanol and polyphenolic compounds; neutral portion B, which contains the flavonols, and acidic phenolics fraction, and the anthocyanin fraction. The significant fractions were examined for anti-microbial activity. every fraction revealed strong antibacterial action, with the exception of the anthocyanin-containing portion. The most delicate among the Gram-positive strains, *Staphylococcus aureus* was present, while among the bacteria that are Gram-negative *Salmonella typhi* was the strain that was most prone to this. Fraction accommodates polyphenols and other flavonols was known for its antimycotic activity. *Trichophyton rubrum* and *Trichophyton mentagrophytes* both showed no signs of growth. The fact that the fractions inhibit certain Aspergillus strains suggests that the compounds in the fractions may be useful in preventing the development of aflatoxins in food products. The antifungal activity of the phenolic acid fraction, which was shown to be the most potent, was further examined against six different fungi, including (*Aspergillus flavus, Penicillium notatum, T. rubrum, Aspergillus niger, T. mentagrophytes, and Microsporum gypseum*). All of the examined fungus species were significantly suppressed by the fraction [64]. *Staphylococcus epidermidis, Bacillus subtilis, Streptococcus pneumoniae, Staphylococcus aureus*, and six Gram-negative strains of *Salmonella typhi* para-A, *Escherichia coli, Proteus vulgaris, Salmonella typhi* para-B, *Shigella dysenteriae,* and *Proteus mirabilis* can all be effectively treated with an ethanol extract of phalsa fruit and bark [88]. Fungal strains Aspergillus fumigatus and Candida albicans as well as 5 bacterial (*Bacillus subtilis, Escherichia coli, Pseudomonas aeruginosa, Klebsiella pneumonia,* and *Staphylococcus aureus*) have been found to be inhibited by methanolic and aqueous root extract [65]. The researchers discovered that a methanolic extract of the phalsa flower (20 mg/ml) reduced the growth of four bacteria: *Pseudomonas aeruginosa* (3.3 0.04 cm), *Escherichia coli, Salmonella abony*, and *Vibrio cholera* (3.0 0.08 cm) [45].

### 5.7 Anti-malarial

It has been demonstrated that methanolic leaf extract possesses antimalarial and antiemetic properties, as stated by Zia-Ul-Haq et al. [61]. The study recommended that phalsa leaves may contain antiemetic and antimalarial medications. The crude methanol extract exhibited 69.0 % suppression of malarial activity. Male chicks were administrated with methanolic extract at doses of 50 mg/kg and 100 mg/kg, and the percentages of emetic action inhibition were 39.15 % and 59.68 %, respectively. Another study by Yaqeen et al. [74] assessment of the antiemetic properties of alcoholic extracts of the fruits of Grewia asiatica in dogs, while acute oral toxicity tests were done on mice and rats. In rats and mice, doses of extract of crude alcohol of 200 mg/kg and 600 mg/kg were shown to be non-toxic. Dogs were given an antiemetic effect from an oral dose of the crude alcoholic extract (120 mg/kg body weight) for 3 h. The centrally acting apomorphine (0.044 mg/kg body weight) produced emesis. This activity was comparable to commonly used commercial anti-emetic drugs such as Largactil (chlorpromazine) and Maxolon (metoclopramide). Apomorphine (0.044 mg/kg body weight) centrally caused emesis. This activity was equivalent to widely used commercial anti-emetic medications such as Maxolon (metoclopramide) and Largactil (chlorpromazine).

### 5.8 Hepatoprotective

The hot and cold fractions of polysaccharide isolated from the phalsa plant have strong hepatoprotective and curative effects; however, the hot fraction containing galacturonic acid has the highest significant protective and curative activity on the hepatotoxicity of CCl4, suggesting that the presence of uronic acids in the polysaccharide complex may enhance the hepatoprotective effect. Liver damaged mice pre-treated with the oral dose of isolated polysaccharide for 7 days with (100 mg/kg bw). In comparison to the standard drugs Silymarin (25 mg/kg) administration of the extract and drug was carried out after liver damage for another 7 days, the conclusion showed that both hot and cold hydrolyzed polysaccharide extracts significantly reduced alanine aminotransferase (hot: 31.2 ± 1.1–39.2 ± 1.3 U/L and cold: 33.4 ± 1.2–47.4 ± 2.1 U/L) and liver aspartate aminotransferase (hot: 36.3 ± 1.2–46.1 ± 2.3 U/L, cold: 38.4 ± 1.6–53.8 ± 2.2 U/L) [69]. Serum AST, ALT, ALP were measured, in addition to protective effects, pre-treatment with various plant extracts reduced the levels of the biochemical markers AST, ALT, and ALP in the groups that received whole ethanol, polysaccharides, ethyl acetate, and aqueous ethanol extracts in liver-damaged rats as compared to silymarin [17,89].

### 5.9 Anti-hyperlipidemic

Phalsa leaves have potent antihyperlipidemic properties (Abou et al., 2005). Induced hyperlipidemic rats were used in the investigation, whose measurements revealed that leaves of phalsa had the result of reducing the level of cholesterol inside manipulated rats. In the study, however, did not evaluate the mode of action or active ingredients indulged. More recently, the study of two animals found that the plant phalsa has antihyperlipidemic properties and also identified the key active ingredients [39,75]. Norwegian rats were administered with the methanolic extract of phalsa plant bark (200 mg/kg and 400 mg/kg bw) for 15 days. This lowers the rats’ density of lipoproteins (76.3 ± 4.32–16.3 ± 2.94 mg/dl), total cholesterol (136.7 ± 14.7–67.3 ± 8.04 mg/dl), very low-density lipoproteins (23.13 ± 1.1–15.4 ± 0.74 mg/dl), and triglycerides. In contrast, more density lipoprotein levels rose in the tested rats (5.7 ± 1.77–34.3 ± 2.01 mg/dl) [39]. Research for aqueous (MeOH) methanolic extract with (30:70) of fresh fruit of the phalsa plants remarkably decreased triglyceride, cholesterol, blood clotting, and low-density lipoprotein (activated partial thromboplastin, prothrombin, fibrinogen, and thrombin) while more density of lipoprotein in hyperlipidemia rats [75]. This finding is consistent with [39].

### 5.10 Analgesic

Using the acetic acid-induced writhing test and Eddy's hot plate test paradigm, the central and peripheral analgesic effects of aqueous and methanolic extracts of phalsa root bark have been assessed. Oral root bark extracts were administered to male Swiss albino mice. According to the results of the acetic acid-induced writhing test model, the central analgesic (Eddy's hot plate test) revealed no result for any treatment and have positive control after 180 min, while the aqueous extract (400 mg/kg bw) had a maximum analgesic effect at 46.24 % in contrast to the methanolic extract (400 mg/kg bw) at 41.14 % and the allusion to drug indomethacin (10 mg/kg bw) at 41.14 [68]. Overall, the study's conclusions showed that both the aqueous and methanolic extracts had a potent analgesic effect. Phalsa fruit extract has shown analgesic efficacy (Debajyoti et al., 2012). Similar to the earlier work, the acetic acid-induced writhing test and hot plate test were allowed to assess analgesic properties. Phalsa fruit aqueous extract given to Swiss albino mice at doses of 100–400 mg/kg bw led to results similar to the positive control.

## 6 Potential industrial applications of phalsa

### 6.1 Functional ingredient in the food industry

Phalsa berries are used as functional food components in the formulation of different products. When ripen, phalsa berries are frequently eaten fresh after a quick wash, in sweets, or after being transformed into different drinks and beverages contributing nutritious value and health-promoting properties and sensory characteristics [90]. Phalsa can be used to prepare various value-added products in order to increase its shelf life, phalsa pickles can be prepared by immersing mature, somewhat unripened entire fruit (green in color) into vinegar or oil. By gently boiling the unripe fruit in sugar syrup, it can also be used to make phalsa preserve. Throughout the summer, phalsa berry-based beverages and fresh juice are highly popular in the local markets of the Indo-Pak Subcontinent. After being separated, the pulp is diluted in water with sugar and salt. The beverage is thought to provide a cooling effect against the humid tropical weather [9].

According to reports, phalsa juice ferments extremely quickly, and the only ways to prevent this are by processing or using artificial preservatives like potassium metabisulphite or sodium benzoate [91]. The latter may additionally systematically have a bleaching impact on the product's color. So, sodium benzoate is suggested as a preservative regarding phalsa goods. Techniques for long-term processing and storage, such as the thermal pasteurization of juice and beverages made with phalsa cause significant reductions in the natural fragrance. This issue can be resolved by utilizing practice of biological aroma release. Phalsa juice is either given an enzyme called glucosidase, or the juice with the enzyme that has been immobilized to increase the content of aroma components like pinenes, terpenes, Linalool, as well as certain lactones linked to another compound. To extract phalsa juice, ripe fruits are washed followed by the addition of water in a 1:0.5 ratio and heating for softening of the fruit. After heating, crushing is done by passing through a pulper which is then strained to separate juice and residual particle. RTS phalsa drink can be prepared by mixing extracted juice with syrup (sugar + water + citric acid) followed by heating to get the proper consistency. The preservative can also be added to increase the shelf life of prepared products, then, juice can be poured/filled in bottles followed by pasteurization for 15 min at 85 °C. Another value-added product that can increase consumer attraction on a sensory basis is carbonated phalsa beverages. Phalsa squash is a different food made from fruit pulp and sugar syrup, the method of preparation is the same as that of RTS phalsa drink, but TSS is maintained at 40–50 %, fruit juice 25 %, and 1.0 % citric acid [92]. Jam made from phalsa leaves, fruit pulp, and sugar can be combined to be used as a topping for baked goods. Phalsa leaves and fruits can be used to make a tasty and healthy herbal tea by boiling them in water [93].

### 6.2 Medicinal applications

In addition to being a food source, Phalsa is also a herbal remedy that can be used to treat a number of ailments. The fruit has been used as a folk remedy as a cooling, stomachic, and astringent agent [62]. It has been claimed that when unripe, it relieves was given to people with respiratory and cardiac, and blood disorders, in addition to fever [94]. Bark and roots have been used as an infusion to treat rheumatism as a demulcent [61]. Leaves were applied to erupted skin problems. Bark was used for the treatment of wounds, ulcers, leprosy, cough, gonorrhea, earache, and headache [62]. The quantity of therapeutic studies conducted on several components of this plant, including the stem, leaves, and fruits. The fruits can also be used to treat various disorders throat, tuberculosis (TB), and sexual impotence., as well as for thirst, anorexia, indigestion, spermatorrhoea, asthma, toxaemia, diarrhea, stomatitis, and hiccough. Additionally, the fruit has a radioprotective effect. Anticancer activity liver and breast cancers are resistant to the aqueous extracts of fruits and leaves. As a result, phalsa fruit and leaf extracts can be used to treat human cancer. Fruit might help with throat issues as well. Phalsa seeds are said to contain abortifacient and anti-implantation properties, acting as an antifertility agent. Its juice can be used to treat diabetes because it has a low glycemic index. Foods with a low glycemic index break down carbohydrates more slowly. Additionally, it is believed that foods with a low glycemic index reduce the incidence of obesity and coronary heart disease [9].

### 6.3 Animal fodder

In dry locations, shrubs are valued as genetic resources for supplemental fodder. Fresh leaves are prized for their nutritional value and composition as animal fodder. One plant can provide around 1.47 kg of green biomass for feeding purposes. The leaves with 12.50% crude protein, 24.09% cellulose, 20.86% lignin, 63.42% NDF, 48.68% ADF, and 8.95% ash based on dry weight content are all considered ideal fodder [20].

### 6.4 Other uses

Leaves of phalsa plants are used as animal fodder. In some places, the bark is utilized as an alternative to soap. To clear sugar and jaggery, the bark's mucilaginous extract is employed. The rope is made from fiber that has been removed from the bark. The wood is fine-grained, yellowish-white, sturdy, and flexible [95]. The pruned branches are employed to make baskets, support sticks, and fuel wood. A long-chain keto alcohol called grewinol is present in the flower. It is used for archer's bow, shingles, poles, and spear handles for carrying the burden on the shoulders [21].

## 7 Current and future prospective

Phalsa fruit has recently demonstrated great potential as a conventional and practical food ingredient for the development of innovative food preserves and beverages. At present, the fruit of the phalsa plant is primarily employed for the formulation of popular goods on a small-scale, even domestic level. Berries of phalsa plant are most frequently grown wild or only for shorter periods it appears in the market due to 1–2 days of life period. For advertising, it is necessary to produce these berries in a more structured way if industrial-scale processing is to be done with them. Fruits of desirable quality may result from this method, which will assist in minimizing berries postharvest losses accomplished keeping in mind its deliberate use. Additionally, the creation of recent cultivars for larger fruits, increased yield, quality, sweetness, and flavor; insect resistance, and ability to cultivate in colder climates. Despite minimal efforts to increase an appropriate cold supply network to ensure supply to far-off markets.

## 8 Conclusion

Phalsa fruit contains several bioactive compounds such as anthocyanin, flavonoids, and tannins, which contribute to its significant functional properties. Moreover, this berry fruit may effectively be utilized in many pharmaceutical applications such as alleviating stomach upset, treating intestinal infection, suppressing cough, managing diarrhea, and addressing jaundice by controlling the glycemic index. Phalsa has physiologically active compounds that have been discovered in many parts of the plant. Research suggests that these components contribute to antioxidant, antidiabetic, radioprotective, antibacterial, anticancer, and anti-hyperlipidemia properties. The limited utilization of phalsa is mostly attributed to its short shelf life and the diminutive size of the phalsa fruit, despite its potential in elucidating the functional importance of many bioactive compounds present in phalsa plants. The potential benefits of Phalsa should not be ignored, despite its potentially lower marketability compared to some other produced commodities. Phalsa is being processed on a small scale to produce many marketable products, including squash, jam, drinks, juice, and syrup. Therefore, there is a need for further studies focused on the phalsa plant, particularly in relation to the utilization of advanced techniques to improve its nutritional composition and overall quality.

## Funding information

National Research, Development and Innovation Fund of Hungary: TKP2022-NKTA-32.

## CRediT authorship contribution statement

**Simrat Kaur:** Writing – original draft, Visualization, Validation, Resources, Methodology, Investigation, Formal analysis. **Rafeeya Shams:** Writing – review & editing, Writing – original draft, Validation, Supervision, Resources, Project administration, Methodology, Investigation, Formal analysis, Data curation. **Kshirod Kumar Dash:** Writing – review & editing, Writing – original draft, Visualization, Validation, Supervision, Software, Resources, Project administration, Methodology, Investigation, Funding acquisition, Formal analysis, Data curation, Conceptualization. **Vinay Kumar Pandey:** Validation, Software, Formal analysis. **Ayaz Mukarram Shaikh:** Writing – review & editing, Visualization, Software, Resources. **Endre Harsányi:** Writing – review & editing, Visualization, Validation, Software, Resources. **Béla Kovács:** Writing – review & editing, Visualization, Validation, Software, Resources.

## Declaration of competing interest

The authors declare that they have no known competing financial interests or personal relationships that could have appeared to influence the work reported in this paper.
